# Editorial: Insights in phage biology: 2021

**DOI:** 10.3389/fmicb.2022.1052366

**Published:** 2022-10-27

**Authors:** Adelaide Almeida

**Affiliations:** Department of Biology and Centre for Environmental and Marine Studies (CESAM), University of Aveiro, Aveiro, Portugal

**Keywords:** bacteriophages, bacteria, resistance, antibiotic, virulence

Antibiotic resistance is an emerging problem that is forcing a growing search for alternative approaches to conventional antibiotics (O'Neill, 2016; Wainwright et al., 2017). Phage therapy emerged as an effective solution that can be used to deal with antibiotic resistance in human, animal, and environmental sectors. Bacteriophages are bacteria-specific viruses that exhibit antimicrobial activity against antibiotic-resistant bacteria without infecting the host cells. They are present in terrestrial and aquatic ecosystems, including plants, animals, and humans, and can be used as a health strategy to combat antibiotic resistance (Garvey, 2020).

The antibiotic resistance crisis motivates the scientific community to rethink the use of bacteriophages to control human infections caused by antibiotic-resistant bacteria. However, despite the early successes, the use of phage therapy in the clinic is sporadic in the Western world, it is only used as a compassionate treatment when antibiotics do not work (Schooley et al., 2017; Dedrick et al., 2019; Law et al., 2019). Contrary to Eastern Europe, in Western Europe and USA, no application currently holds approval that allows phage therapy free practice in the treatment of human infections.

Although phage therapy has been used as a compassionate treatment when antibiotics do not work, antibiotic resistance is not the only problem. There are many other infections that require urgent development, such as wounds, intracellular bacterial infections, infections caused by biofilm-forming bacteria, and bacterial chronic infections, for which antibiotics are not effective. For these indications, phages are ideal even when bacteria are antibiotic-sensitive.

Bacteriophages have also been used as promising candidates for controlling bacterial infection in other areas, such as in veterinary medicine, the food industry, agriculture, and aquaculture (Fernández et al., 2018; Schulz et al., 2022). In these cases, it is imperative to evaluate the impact of bacteriophages on the environment and also the effect of abiotic factors on phage viability; for instance temperature, pH, salinity, and radiation (Pereira et al., 2021, 2022).

Furthermore, overall, more *in vivo* studies are also required to translate the approach to the field when phage therapy is used in humans, animals, or the environment. *In vitro* studies are not plentiful to comprehend phage-bacteria interactions that go on *in vivo*, which hinders the translation of the treatment to the field (Park and Nakai, 2003; Silva et al., 2014, 2016).

Another important aspect to consider when bacteriophages are used is the presence of virulent genes in the phage genomes, such as antimicrobial resistance genes and genes codifying for toxins, potentially influencing host performance, namely its virulence. This phenomenon is more frequent in temperate phages (Silveira et al., 2020; de Nies et al., 2021), not used in phage therapy. However, some lytic phages can integrate their nucleic acid into the host genome, becoming prophages. Several studies have shown that the presence of prophages can affect host characteristics, in particular, antibiotic tolerance, biofilm formation ability, and toxicity (Fortier and Sekulovic, 2013; Touchon et al., 2017; Costa et al., 2018).

In this special issue, recent developments in some of these phage treatment topics are pointed out. It comprises four papers focused on the use of bacteriophages in humans. The first two involve the evaluation of the use of phages on the human microbiota (Buttimer et al.); in animals (Li et al.), including *in vitro* and *in vivo* studies. The second two papers are focused on the study of new phages, belonging to new clusters, which help to understand the virus-host interaction (Dong et al.) and the implication of the presence of genes codifying for virulent factors, namely toxins, in phage genomes (Ribeiro et al.).

As regards the use of phage therapy in humans (Buttimer et al.), the results of the study show that a cocktail of six phages targeting two bacteria is implicated in the development of the human gastrointestinal tract (IBD). *Escherichia coli* and *Enterococcus faecalis*, used as part of a defined community of a bacterial consortium with six members (SIHUMI-6), caused a 1.1–1.5 log reduction in the bacterial numbers when tested in a continuous fermenter, but not when tested in a murine colitis model. The phage addition in the fermenter cause variations in other SIHUMI-6 bacterial strains, increasing *Lactobacillus plantarum* and *Faecalibacterium prausnitzii*. The results obtained using the mice model also suggest that the phage cocktail impacted the other SIHUMI-6 consortium, but did not affect the target bacteria. Overall, it can be concluded that the potential of phages to reduce host populations *in vitro* did not translate to an *in vivo* model, which has implications for translation to the field and the use of phages as a tool to engineer microbial populations (Buttimer et al.). In the study involving the use of phages on animals (Li et al.), 15 newly isolated phages targeting *Salmonella*, the most common pathogen in poultry, but also a significant foodborne pathogen, exhibited lytic capacities and infected a wide host range of serovars of *S. enterica in vitro*. Three of these phages were used in a cocktail and tested 24 h before and during for *Salmonella* and compared to *S*. Enteritidis in a chicken model. The treatment significantly reduced intestinal tract colonization and decreased inflammation in the duodenum. The results indicate that the three phages cocktail could be used against multidrug-resistant *Salmonella* in animal production, mitigating the transmission of infections by zoonotic *Salmonella* species. The *in vitro* results were confirmed in the *in vivo* studies, but, taking into account the results obtained in the study of Buttimer et al., more studies, such as histology scores of the human gastrointestinal tract of the chicken model, should be done in order to translate the treatment to the field.

In the study of Dong et al., a novel lytic *Shewanella* phage, with a linear double-strand genome, was isolated from the surface coastal waters. It was the first isolated Podovirus infecting *Shewanella* and the phylogenetic analysis suggests that this virus can represent a novel viral genus, *Bocovirus*. As in this study, most of the phages used to inactivate bacteria are isolated from the environment (Dong et al.), including adverse environments. Environmental selection pressures and the survival of the fittest generates a rich collection of competitive phage types in the environment. This study reports a new interaction system that can be used for studying phage–host interactions, providing new genome information for viral metagenomic analysis in the marine environment.

Although phages have been indicated as achievable solutions for controlling bacterial diseases, lytic phages can become prophages, influencing host characteristics. In the study of Ribeiro et al., all complete genomes of *Paenibacillus larvae* (the agent of American Foulbrood, a bacterial disease that affects honeybee brood worldwide) available on the NCBI database, are evaluated in order to detect prophages. The 55 prophages identified in the genomes of the 11 *P. larvae* were examined for the existence of genes encoding relevant characteristics related to *P. larvae*, such as antimicrobial resistance, toxins, or bacteriocins activity. This is the first in-depth study of *P. larvae* prophages showing significant knowledge of their impact on the host virulence and fitness in the five ERIC genotypes of *P. larvae*, as new features were assigned to the viruses. Although more studies are necessary in order to confirm these results, using, for instance, a higher representation of *P. larvae* strains diversity, the results show the relevance of the role of prophages in the pathogeny of *P. larvae*, regarding its virulence and fitness.

Overall, these four papers gathered more knowledge of phage treatment, demonstrating that bacteriophages are promising tools for the control of pathogenic bacteria in different sectors, but there is still a need for new developments to translate this technology to the field, such as more *ex vivo* and *in vivo* studies.

It was a pleasure to edit a special scientific publication with the results of these important recent studies from different research groups to inspire researchers to respond to the current underutilization of phages.

## Author contributions

The author confirms being the sole contributor of this work and has approved it for publication.

## Funding

The author acknowledges the financial support to CESAM by FCT/MCTES (UIDP/50017/2020+UIDB/50017/2020+ LA/P/0094/2020) through national funds.

## Conflict of interest

The author declares that the research was conducted in the absence of any commercial or financial relationships that could be construed as a potential conflict of interest.

## Publisher's note

All claims expressed in this article are solely those of the authors and do not necessarily represent those of their affiliated organizations, or those of the publisher, the editors and the reviewers. Any product that may be evaluated in this article, or claim that may be made by its manufacturer, is not guaranteed or endorsed by the publisher.

## References

[B1] Costa A. R. Monteiro R. Azeredo J. (2018). Genomic analysis of Acinetobacter baumannii prophages reveals remarkable diversity and suggests profound impact on bacterial virulence and fitness. Sci. Rep. 8, 1–11. 10.1038/s41598-018-33800-5 30337588 PMC6193963

[B2] de Nies L. Lopes S. Busi S. B. Galata V. Heintz-Buschart A. Laczny C. C.. (2021). PathoFact: a pipeline for the prediction of virulence factors and antimicrobial resistance genes in metagenomic data. Microbiome 9, 1–14. 10.1186/s40168-020-00993-9 33597026 PMC7890817

[B3] Dedrick R. M. Guerrero-bustamante C. A. Garlena R. A. Daniel A. Ford K. Harris K.. (2019). Engineered bacteriophages for treatment of a patient with a disseminated drug resistant Mycobacterium abscessus. Nat. Med. 25, 730–733. 10.1038/s41591-019-0437-z 31068712 PMC6557439

[B4] Fernández L. Gutiérrez D. Rodríguez A. García P. (2018). Application of bacteriophages in the agro-food sector: a long way toward approval. Front. Cell. Infect. Microbiol. 8, 1–5. 10.3389/fcimb.2018.00296 30186776 PMC6113595

[B5] Fortier L. C. Sekulovic O. (2013). Importance of prophages to evolution and virulence of bacterial pathogens. Virulence 4, 354–365. 10.4161/viru.24498 23611873 PMC3714127

[B6] Garvey M. (2020). Bacteriophages and the one health approach to combat multidrug resistance: is this the way? Antibiotics 9, 1–17. 10.3390/antibiotics9070414 32708627 PMC7400126

[B7] Law N. Logan C. Yung G. Furr C. L. L. Lehman S. M. Morales S.. (2019). Successful adjunctive use of bacteriophage therapy for treatment of multidrug-resistant Pseudomonas aeruginosa infection in a cystic fibrosis patient. Infection 47, 665–668. 10.1007/s15010-019-01319-0 31102236

[B8] O'Neill J. (2016). Tackling Drug-Resistant Infection Globally: Final Report and Reccommendations. London: AMR Review.

[B9] Park S. C. Nakai T. (2003). Bacteriophage control of *Pseudomonas plecoglossicida* infection in ayu *Plecoglossus altivelis*. Dis. Aquat. Org. 53, 33–39. 10.3354/dao053033 12608566

[B10] Pereira C. Costa P. Pinheiro L. Balcão V. M. Almeida A. (2021). Kiwifruit bacterial canker: an integrative view focused on biocontrol strategies. Planta 253, 1–20. 10.1007/s00425-020-03549-1 33502587

[B11] Pereira C. Duarte J. Costa P. Braz M. Almeida A. (2022). Bacteriophages in the control of Aeromonas sp. in aquaculture systems: an integrative view. Antibiotics 11, 1–33. 10.3390/antibiotics11020163 35203766 PMC8868336

[B12] Schooley R. T. Biswas B. Gill J. J. Hernandez-Morales A. Lancaster J. Lessor L.. (2017). Development and use of personalized bacteriophage-based therapeutic cocktails to treat a patient with a disseminated resistant Acinetobacter baumannii infection. Antimicrob. Agents Chemother. 61, 1–14. 10.1128/AAC.00954-17 28807909 PMC5610518

[B13] Schulz P. Pajdak-Czaus J. Siwicki A. K. (2022). *In vivo* bacteriophages' application for the prevention and therapy of aquaculture animals—chosen aspects. Animals 12, 1–21. 10.3390/ani12101233 35625078 PMC9137707

[B14] Silva Y. J. Costa L. Pereira C. Mateus C. Cunha Â. Calado R.. (2014). Phage therapy as an approach to prevent *Vibrio anguillarum* infections in fish larvae production. PLoS ONE 9, 1–23. 10.1371/journal.pone.0114197 25464504 PMC4252102

[B15] Silva Y. J. Moreirinha C. Pereira C. Rocha R. J. M. Cunha Â. Gomes N. C. M.. (2016). Biological control of *Aeromonas salmonicida* infection in juvenile Senegalese sole (*Solea senegalensis*) with Phage AS-A. Aquaculture 450, 225–233. 10.1016/j.aquaculture.2015.07.025

[B16] Silveira C. B. Coutinho F. H. Cavalcanti G. S. Benler S. Doane M. P. Dinsdale E. A.. (2020). Genomic and ecological attributes of marine bacteriophages encoding bacterial virulence genes. BMC Genom. 21, 1–11. 10.1186/s12864-020-6523-2 32024463 PMC7003362

[B17] Touchon M. Moura de Sousa J. A. Rocha E. P. (2017). Embracing the enemy: the diversification of microbial gene repertoires by phage-mediated horizontal gene transfer. Curr. Opin. Microbiol. 38, 66–73. 10.1016/j.mib.2017.04.010 28527384

[B18] Wainwright M. Maisch T. Nonell S. Plaetzer K. Almeida A. Tegos G. P.. (2017). Photoantimicrobials—are we afraid of the light? Lancet Infect. Dis. 17, 49–55. 10.1016/S1473-3099(16)30268-7 27884621 PMC5280084

